# What do standard radiography and clinical examination tell about the shoulder with cuff tear arthropathy?

**DOI:** 10.1186/1749-799X-6-1

**Published:** 2011-01-05

**Authors:** Bart Middernacht, Philip Winnock de Grave, Georges Van Maele, Luc Favard, Daniel Molé, Lieven De Wilde

**Affiliations:** 1 Ghent University Hospital, De Pintelaan 185, Ghent B-9000, Belgium; 2 University of Tours, Boulevard Tonnellé 10, BP 3223, 37032 Tours Cedex 1, France; 3 Clinic for Traumatology and Orthopaedics, Rue Hermitte 49, 54000 Nancy, France

## Abstract

**Background:**

This study evaluates the preoperative conventional anteroposterior radiography and clinical testing in non-operated patients with cuff tear arthropathy. It analyses the radiological findings in relation to the status of the rotator cuff and clinical status as also the clinical testing in relation to the rotator cuff quality. The aim of the study is to define the usefulness of radiography and clinical examination in cuff tear arthropathy.

**Methods:**

This study analyses the preoperative radiological (AP-view, (Artro-)CT-scan or MRI-scan) and clinical characteristics (Constant-Murley-score plus active and passive mobility testing) and the peroperative findings in a cohort of 307 patients. These patients were part of a multicenter, retrospective, consecutive study of the French Orthopaedic Society (SOFCOT-2006). All patients had no surgical antecedents and were all treated with prosthetic shoulder surgery for a painful irreparable cuff tear arthropathy (reverse-(84%) or hemi-(8%) or double cup-bipolar prosthesis (8%)).

**Results:**

A positive significancy could be found for the relationship between clinical testing and the rotator cuff quality; between acromiohumeral distance and posterior rotator cuff quality; between femoralization and posterior rotator cuff quality.

**Conclusion:**

A conventional antero-posterior radiograph can not provide any predictive information on the clinical status of the patient.

The subscapular muscle can be well tested by the press belly test and the teres minor muscle can be well tested by the hornblower' sign and by the exorotation lag signs.

The upward migration index and the presence of femoralization are good indicators for the evaluation of the posterior rotator cuff.

An inferior coracoid tip positioning suggests rotator cuff disease.

## Background

Painful cuff tear arthropathy (CTA) affects the independence of the elderly [1,2] by altering the biomechanics [3] and bony characteristics of the normal glenohumeral joint [4,5]. CTA is a progressive disease which presents a unique therapeutical challenge necessitating an algorithm for treatment based on clinical and radiological parameters [6].

The seriousness of the disease is evaluated clinically and radiologically.

The Constant and Murley score [7] is a well accepted clinical method to evaluate pain, activities of daily living, passive motion, and active motion. Clinical lag signs seem to have an important predictive value in the assessment of the location and the size of the tear [8]. Plain radiographs are known, since longtime [9], to be a sensitive diagnostic tool to evaluate rotator cuff disorders. A conventional antero-posterior radiograph of the shoulder is the most frequently performed examination to study structural bony wear in CTA [2,10-18]. These structural changes include a small or absent acromio-humeral distance [17,18], an ascension and/or medialization of the center of rotation of the glenohumeral joint [6,17], a femoralization of the proximal humerus [6,19], an acetabularization of the acromion [11], an excavation or thinning of the acromion [11] and medial erosion of the glenoid [16]. The extent of this bony wear seems to be related to the seriousness of the disease [20,21]. These AP-views are also useful to evaluate some morphological osseous properties of the shoulder predisposing to rotator cuff disease: coracoid tip positioning in the lower half of the glenoid may suggests an antero-superior rotator cuff tear [15]; a lateral acromion angle below 70 degrees suggests a full thickness rotator cuff tear [10]; a glenoid inclination angle is bigger (98.6°) in patients having full thickness rotator cuff tears compared to the normal inclination angle (91°) [12] and a large lateral extension of the acromion appears to be associated with full thickness tearing of the rotator cuff [14,22].

Scarce information exists about the relationships between the radiological findings, the clinical evaluation [6,8,21,23,24] and the location and extent of the rotator cuff tear [10,13-15,20]. Nevertheless all these properties have therapeutical consequences either conservative or surgical [6,23,25,26].

To evaluate these relationships the authors hypothesized first that a low Constant score [7] in CTA is an indicator for important bony structural changes as seen on conventional antero-posterior radiographs as mentioned above. Second, lag signs [8] reflect the location of the tendinous tear and the muscular quality. Third, the bony structural changes are a reflection of the location and size of the rotator cuff tear. Fourth, the morphological osseous properties, as mentioned above, are indicative for the location and/or size of the rotator cuff tear.

## Methods

Being part of the multicentrical (Lyon; Reims, Zurich, Lille, Nice, Tours, Ghent, Nancy and Toulouse) group asked by the "Société Française de Chirurgie Orthopédique et Traumatologique" to evaluate eccentric omarthrosis, the authors had access to the preoperative clinical and radiological data and peroperative findings of a cohort of 307 patients treated with a shoulder prosthesis. All of these patients had a standard radiograph in neutral rotation as used in daily practice, 187 of them had a CT-scan and 31 had an MRI-scan.

All data was filled in on uniform charts by the responsible surgeons, collected and turned into one big database. Not all charts were filled in completely explaining all the different numbers of patients (n) used in our study.

**Figure 1:**
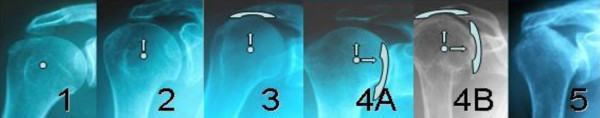
**Hamada's classification of omarthrosis **[11].

The authors studied eccentric omarthrosis, according to the classification of Hamada [11] (figure 1), and centered omarthrosis, with irreparable rotator cuff disease, in patients without any surgical antecedents.

The data on fatty degeneration was derived from CT or MRI-scans with or without arthrography, interpreted by each of the responsible surgeons, taken in the transversal and sagittal plane of the shoulder. The degree of fatty degeneration of the rotator cuff was determined according to Goutallier [27] and the muscular status of the teres minor was defined as normal, hypotrophic, absent or hypertrophic. All patients were divided into two groups for comparison: one with good to acceptable muscular quality (stade 0, 1 and 2 according to Goutallier and normal or hypertrophic) and one with bad muscular quality (stade 3 and 4 and absent or hypotrophic).

The state of the tendons of the rotator cuff is obtained from arthro CT- or MRI-scan and/or peroperative findings, interpreted by the responsible surgeon. The tendons are classified as normal and partially or completely ruptured. All patients were divided into two groups for comparison: one with good to acceptable tendon quality (without rupture) and one group with bad tendon quality (partial or complete rupture).

The clinical evaluation is done according to Constant-Murley [7] (for pain, activities of daily living, range of movement and power); the range of motion of the active external rotation in adduction and abduction; the presence of a hornblower' sign [21] and the feasibility of the press-belly test [28].

**Figure 2:**
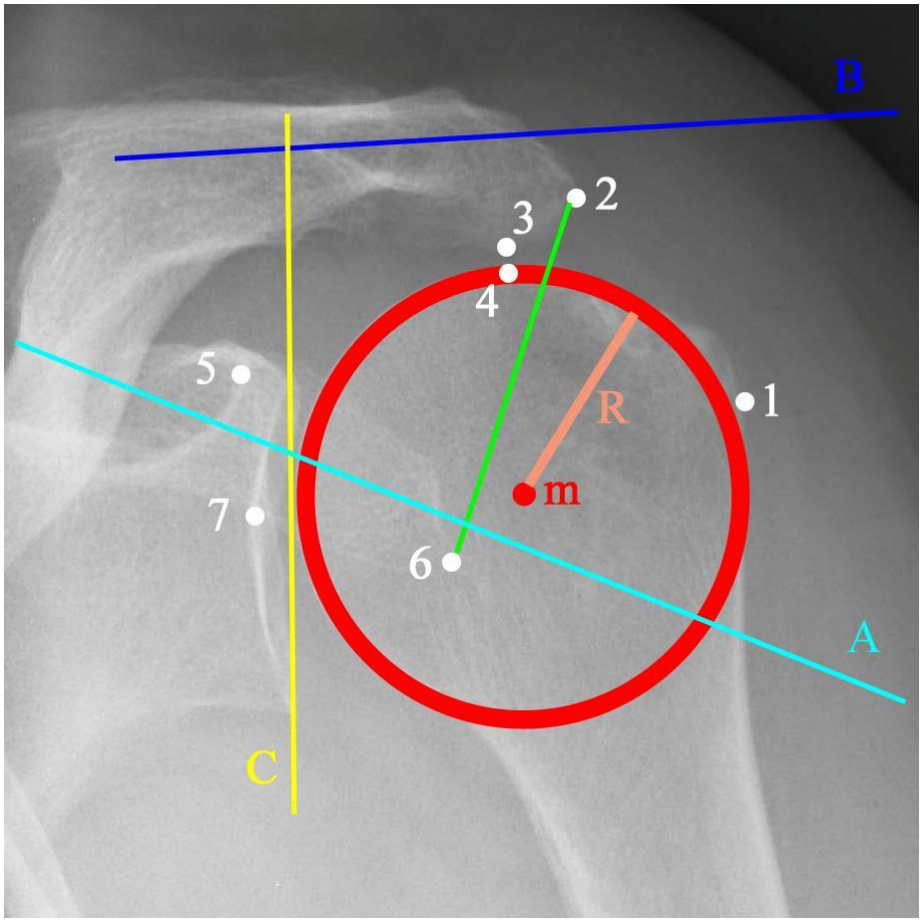
**Example of the marking points and lines drawn onto each radiograph**.

The radiological data, digitally measured by the first author (Adobe^® ^Photoshop^® ^7.0; San Jose, California, US), from patients in a standing position, was obtained on AP-views according to Neer [19] in neutral rotation (Figure 2).

### On AP-view the following marking points were placed (Figure 2)

m: the midpoint of the best fitting circle of the humeral head; 1: the most lateral point of the humeral head; 2: the most lateral point of the acromion; 3: the most inferior point of the acromion; 4: the most superior point of the humeral head; 5: the most lateral point of the coracoid basis; 6: the most lateral point of the coracoid tip.

On AP-view the following lines were placed and their angulations to the horizon were measured (Figure 2):

A: a line best fitting the direction of the coracoid process; B: a line best fitting the direction of the acromion; C: a line best fitting the direction of the glenoid; D: a line connecting marking point 2 and 6.

### On AP-view the following parameters were measured

*humeral head radius: *the radius, in mm, of the best fitting circle of the humeral head.

*acromiohumeral distance*: measured in mm between two lines drawn through point 3 and 4 parallel to the B-line [13,17,29];

*acromial thickness*: measured in mm at the most thin part;

*medialisation and ascention: *measured in mm between marking points m and 5, measured between m and a line parallel with B drawn through point 5 and measured between m and a line parallel with C drawn through point 5 (Figure 2). The distance between point m and the D-line was also measured. The upward migration index [17] was calculated;

*coracoid tip positioning: *the distance between two parallel lines drawn through the most inferior point of the coracoid tip and the most inferior point of the glenoid, parallel to the B-line, compared to the supero-inferior length of the glenoid;

*the mean lateral acromion angle *[14,22] was determined by the difference in degrees between the B- and C-line.

*the glenoid inclination angle *[12] was here determined in relation to the horizontal.

*the acromial index *[14]: the distance from the glenoid plane to the lateral border of the acromion was divided by the distance from the glenoid plane to the lateral aspect of the humeral head.

### On AP-view the following parameters were described

*Femoralization *of the proximal humerus [6,19] was defined as absence or presence of erosion of the greater tuberosity.

*Acetabularization *of the acromion [11] was defined as absent or present.

**Figure 3:**
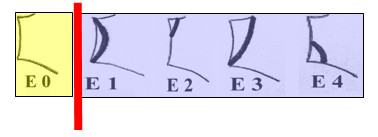
**The classification of Sirveaux et al**. [16]** was used to devide the glenoids into two groups**.

*Medial erosion of the glenoid *was defined as absent (E0) or present (E1, E2, E3 and E4) according to Sirveaux et al. [16] (figure 3).

The relationships between the different clinical parameters as well as the total Constant score and all radiological parameters cited above are analysed.

Statistical analysis was performed with R (a language and environment for statistical computing) [30].

Univariate comparison was done with the Fisher's Exact test for categorical data. The non-parametric Mann-Whitney U-test was used to compare continuous variables. Also the Spearman correlation was used. The significance level was set at alpha = 0.05.

Five different radiographs were analysed twice by the first author in order to determine the intra-observer variability. There was only one observer so an inter-observer variability was not to be performed. To determine these variabilities, the intraclass correlation coefficient was used (ICC), in combination with the Wilcoxon Signed Ranks test [31].

## Results

### Descriptive measurements

According to Hamada [11] we defined 25 patients as type 1, 53 patiens as type 2, 27 patients as type 3, 48 patients as type 4a, 94 patients as type 4b, 27 patients as type 5 and 33 patients as centered omarthrosis (Figure 1).

On CT- or MRI- (arthro-)scan the infraspinate muscle is fatty degenerated for at least half of its volume in 82% of described cases; the subscapular muscle in 49% of patients and the teres minor muscle was atrophic or absent in 32% of described patients.

On arthro CT- or MRI-scan and peroperative findings, the supraspinate tendon is partially or completely ruptured in 98% of described cases; the infraspinate tendon in 69% of cases; the subscapular tendon in 92% of cases and the teres minor muscle in 37% of described patients.

The mean Constant-Murley score is 24/100 (10) (mean (SD)) (n = 307).

The mean acromiohumeral distance is 4.5 mm (3.6). The mean humeral head radius is 24 mm (5). The mean acromial thickness is 6.2 mm (2.5) and the mean lateral extension of the acromion is 9.8 mm (6.0). The mean supero-inferior distance of the glenoid is 36 mm (7). We defined 240/294 (82%) of our patients to be type I coracoid according to Schulz et al. [15]

The Intraclass Correlation Coefficient [31] was 0,982 (95% confidence interval (CI): 0.875, 0.998).

*Relationships between bony structural changes versus Constant score are summed up in *table 1.

**Table 1:** Relationships between bony structural changes versus Constant score.

**table 1 evaluation of the statistical relationship between the Constant score and:**	**statistical test used**	**P-value to evaluate significance (number of cases)**
Acromio-Humeral distance	Pearson	0,377 (305)

Medialisation	Spearman	0,064 (303)

Femoralisation	Mann-Whitney U	0,315 (281)

Acetabularisation	Mann-Whitney U	0,966 (303)

Acromial thickness	Mann-Whitney U	0,099 (303)

Medial erosion of the glenoid	Mann-Whitney U	0,653 (303)

*Relationships between lag signs versus location of the tendinous tear and muscular quality can be seen in *table 2.

**Table 2:** Relationships between lag signs, bony structural changes and morphological osseous properties versus location of the tendinous tear and muscular quality.

P-Values calculated with the Fisher's exact statistical test between colum and row (number of cases)	**Subscapular muscular quality**	**Infraspinatus muscular quality**	**teres minor muscular quality**	**Subscapular tendon tear**	**Supraspinatus tendon tear**	**Infraspinatus tendon tear**	**teres minor tendon tear**
exorotation in adduction	0,16 (166)	0,113 (167)	**<0,001 (137)**	**0,05 (234)**	1 (208)	1 (208)	**0,003 (121)**

exorotation in abduction	0,367 (88)	1 (89)	**<0,001 (76)**	0,834 (123)	0,519 (100)	1 (100)	0,052 (66)

hornblower's sign	0,547 (103)	0,092 (65)	**0,004 (45)**	0,432 (76)	0,548 (72)	0,548 (72)	**0,002 (55)**

press belly test	**<0,001 (111)**	1 (110)	0,82 (100)	**<0,001 (132)**	0,247 (118)	0,503 (119)	0,387 (94)

Upward migration index	0,305 (231)	**0,019 (230)**	**0,029 (190)**	0,373 (304)	0,665 (277)	**0,012 (278)**	0,794 (170)

Medialisation	0,281 (231)	0,59 (230)	0,332 (190)	0,705 (304)		0,253 (278)	0,252 (170)

Femoralisation	0,519 (231)	**<0,001 (230)**	**<0,001 (190)**	**0,042 (304)**	0,496 (277)	**0,003 (278)**	**<0,001 (170)**

Medial erosion of the glenoid	0,293 (231)	0,165 (230)	**0,029 (190)**	**0,024 (303)**	0,66 (276)	0,65 (277)	0,428 (170)

Acetabularisation	0,684 (231)	**0,018 (230)**	0,419 (190)	1 (304)	1 (277)	0,164 (278)	0,141 (170)

lateral acromion angle	0,277 (231)	0,774 (230)	0,796 (190)	0,69 (304)	1 (277)	1 (278)	0,793 (170)

acromial index	0,28 (231)	0,474 (230)	0,339 (190)	0,256 (304)	0,653 (277)	0,647 (278)	0,113 (170)

acromial thickness	0,084 (231)	0,076 (230)	0,756 (190)	0,901 (304)	1 (277)	0,819 (278)	0,526 (170)

Glenoid inclination angle	0,185 (231)	0,052 (230)	0,347 (190)	0,172 (304)	0,665 (277)	0,068 (278)	0,341 (170)

*Relationships between the location of the tendinous tear and muscular quality of the rotator cuff versus bony structural changes and morphological osseous properties are also displayed in *table 2.

## Discussion

An anteroposterior radiograph is used today to document patients with rotator cuff tear arthropathy. Furthermore this basic investigation is applied to distinguish various types of the disease with specific therapeutical consequences.

This multicenter database studies preoperative conventional anteroposterior radiographs, in non-operated patients with cuff tear arthropathy, in relation to the clinical status and the status of the rotator cuff derived from peroperative findings, CT- and MRI-scans.

Being multicenter will be the major weakness of this study because nine different institutes provided the clinical data and peroperative findings. However, to our knowledge no such study exists evaluating these relationships on such an important number of patients (n = 307).

Another weakness of this study is that we didn't have a CT and/or MRI for each patient. However we did have a large number of CT's and MRI's and had peroperative findings for each of the patients. The last minor point of our work will be the lack of a control group without cuff tear arthropathy.

The seriousness of clinical impairment of our studied population is reflected by the low mean Constant score (24/100). Because this study could not find any relationship between the radiologic extent of the bony structural changes and the clinical status of the patient, we believe a conventional antero-posterior radiograph cannot provide any predictive information on the clinical status of the patient. This is in contrast with the statement of Nové-Josserand et al. who demonstrates a strong statistical correlation between the Constant score versus Hamada stage or the severity of the glenohumeral degradation [2].

We agree with Tokish et al. who found the subscapular muscle and tendon can be well tested with the press belly test [28] and with Walch et al. who stated the teres minor muscle and tendon can be well evaluated with the hornblower' sign [21]. Our study also confirms the statement of Hertel et al. who found clinical testing for lag signs to be efficient, reproducible, and reliable in evaluating the teres minor tendon and muscle [8].

We found the upward migration index [18] and the presence of femoralization [6,19] to be good indicators for the evaluation of the posterior rotator cuff. Therefore we can agree with van de Sande et al. [18] who stated that fatty infiltration of the infraspinatus muscle shows the strongest correlation with proximal migration.

We could not find any significant relationship between the rotator cuff status on the one hand and medialization, vertical erosion of the glenoid [16] and acetabularization [11] on the other hand. This relativates the statement of Visotsky et al. who suggests that the amount of decentralization depends on the extent of the rotator cuff tear, the integrity of the coracoacromial arch, and the degree and direction of the glenoid bone erosion [6].

All our studied patients had rotator cuff disease and 82% of them had an inferior projection to the middle of the glenoid (type I coracoid tip positioning) [15]. We could not find a visible difference in coracoid tip positioning and site of the rotator cuff weakness as proposed by Schulz et al. [15] who concluded that type I coracoids are predominant in shoulders with supraspinatus tears and type II coracoids in shoulders with subscapularis tears.

Furthermore we could not find any significant relationship between the location and/or site of the rotator cuff tear versus the lateral acromion angle [10], the acromion index [14,22] and the glenoid inclination angle [12]. These three latter morphological osseous properties are predictive for the general rotator cuff quality [10,14,22] but are of less use in localizing the cuff tears.

## Conclusions

A conventional antero-posterior radiograph cannot provide any predictive information on the clinical status of the patient.

The subscapular muscle can be well tested by the press belly test [28].

The teres minor muscle can be well tested by the hornblower' sign [21] and by the exorotation lag signs [8].

The upward migration index [18] and the presence of femoralization [6,19] are good indicators for the evaluation of the posterior rotator cuff.

82% of patients with rotator cuff disease present with an inferior coracoid tip positioning to the glenoid [15].

## Competing interests

The authors declare that they have no competing interests.

## Authors' contributions

BM: Collecting data; analysing data; writing the article; PWdG: Collecting data;

GVM: Statistical analyses; LF: Providing data; DM: Providing data;

LDW: Coordinating; providing data; providing study idea; writing the article.

All authors have read and approved the final manuscript.
